# Reduced expression of miR-22 in gastric cancer is related to clinicopathologic characteristics or patient prognosis

**DOI:** 10.1186/1746-1596-8-102

**Published:** 2013-06-21

**Authors:** Weibin Wang, Fujun Li, Yong Zhang, Yanyang Tu, Qi Yang, Xingchun Gao

**Affiliations:** 1 Department of General Surgery, The 323th Hospital of PLA, Xi’an 710054, China; 2 Authorities outpatient Department of Lanzhou Military region, Lanzhou 730000, China; 3 Department of Experimental Surgery, Tangdu Hospital, Fourth Military Medical University, Xi’an City 710038, P.R. China

**Keywords:** MicroRNA-22, Gastric cancer, Prognosis, Quantitative RT-PCR

## Abstract

**Objective:**

Involvements of microRNA-22 (miR-22) in cancer development have attracted much attention, but its role in tumorigenesis of gastric cancer is still largely unknown. Therefore, the aim of this study was to investigate the expression patterns and clinical implications of miR-22 in gastric cancer.

**Methods:**

Quantitative RT-PCR was performed to evaluate the expression levels of miR-22 in 98 pairs of gastric cancer and normal adjacent mucosa.

**Results:**

Compared with normal adjacent mucosa, miR-22 expression was significantly downregulated in gastric cancer tissues (P < 0.001). Of 98 patients with gastric cancer, 58 (59.2%) were placed in the low miR-22 expression group and 40 (40.8%) were placed in the high miR-22 expression group. In addition, tumors with low miR-22 expression had greater extent of lymph node metastasis (P = 0.02) and distant metastasis (P = 0.01), and were at a worse stage (P = 0.01) than the tumors with high miR-22 expression. Moreover, the gastric cancer patients with low miR-22 expression had shorter overall survival than those with high miR-22 expression (P = 0.03). MiR-22, determined by multivariate analysis, was an independent prognostic factor for patients with gastric cancer.

**Conclusion:**

Our data offer the convincing evidence that the reduced expression of miR-22 was significantly associated with malignant development of gastric cancer and may be a novel prognostic marker of this disease. miR-22 might have potentials in the application of cancer therapy for patients with gastric cancer.

## Introduction

Gastric cancer is the fourth most prevalent forms of human cancers and the second leading cause of cancer-related death in the world, especially in East Asian countries. Its incidence rate is 20 per 100,000 annually [1]. According to its histological subtypes, gastric cancer can be divided into two groups: the intestinal type and the diffuse type. The former is characterized by expansive growth and liver metastasis; whereas the latter is characterized by infiltrative growth and peritoneal dissemination [2-4]. They are both associated with Helicobacter pylori infection that contributes to more than 80% of cases [5]. Nowadays, gastrectomy remains the mainstay treatment for gastric cancer, but the prognosis for advanced stage patients is still very poor. The median survival time for patients with gastric cancer is only 6–9 months [6]. In China, the 5-year overall survival rate of patients with gastric cancer is lower than 40%, although recent advances in chemotherapy and surgical techniques [7]. This is primarily attributed to the following reasons: lack of diagnostic markers for early detection, weak prognostic value of histological indicators, limited efficiency of current treatment for advanced disease and lack of molecular markers utilized for targeted therapy [8-10]. Therefore, it is of great significance to make a better understanding of gastric carcinogenesis and to identify novel molecular markers for the improvement of clinical management of patients with gastric cancer.

MicroRNAs (miRNAs) are a recently discovered category of small (19 ~ 24 nucleotides), non-protein-coding and single-stranded endogenous RNA molecules [11]. miRNAs function as regulators of approximately 60% protein-coding genes’ expression mainly at the post-transcriptional level by binding to the sequences in the 3′ untranslated regions (3′-UTR) of their targeted mRNAs resulting in translational repression or gene silencing [12]. As they are involved in regulation of wide array of biological processes including cell proliferation, differentiation, apoptosis, metastasis, angiogenesis and immune response, miRNAs have been considered to be new approaches of tumor biomarkers for early cancer diagnosis and prognosis. They may play roles in the development and progression of cancers similar to those played by oncogenes or tumor suppressor genes. Recent studies have identified a number of miRNAs with aberrant expression in gastric cancer. For example, the comparison of miRNAs deregulated in gastric cancer revealed a significant increase of several tumor-associated miRNAs such as miR-21, -25 and -106a and miRNAs from the miR-17-92 cluster [13]; based on the cluster analyses, eight miRNAs (including miR-100, -143 and −145) were upregulated specifically in diffuse-type, while four miRNA (miR-202, -373, -494 and −498) in intestinal-type gastric cancer [14]. In our previous study, we found that the downregulation of miR-206 was significantly correlated with tumor progression and may be a potent prognostic marker of gastric cancer [15]. According to our literature retrieval, miR-22 has been demonstrated to play important roles in different types of cancer, such as hepatocellular carcinoma, breast cancer, colon cancer, lung cancer, and prostate cancer [16-22]. However, its roles in tumorigenesis of gastric cancer are still unknown. Because of its involvement in several tumors in digestive system (hepatocellular carcinoma and colon cancer), we hypothesized that miR-22 might play a role in gastric cancer. Thus, the aim of the present study was to investigate the expression patterns and clinical implications of miR-22 in gastric cancer.

## Materials and methods

### Patients and tissue samples

This study was approved by the Research Ethics Committee of the 323th Hospital of PLA and Tangdu Hospital of the Forth Military Medical University, China. Written informed consent was obtained from all patients. All specimens were handled and made anonymous according to the ethical and legal standards.

Ninety-eight pairs of gastric cancer and matched normal adjacent mucosa were resected from gastrectomy with lymph node dissection between 1999 and 2007 at Department of General Surgery. These patients with gastric cancer included 62 males and 36 females, ranged in age from 21 to 86 years (mean 63 years). Clinicopathologic findings were based on the criteria of the tumor node metastasis (TNM) classification of the International Union against Cancer [23]. Histopathological types of gastric cancer were classified into two types, intestinal type and diffuse type. The intestinal type was further classified into three differentiated types: well-differentiated (tub1), moderately differentiated (tub2), and papillary differentiated (pap). The diffuse type was classified into two undifferentiated types: diffuse-adherent (por1) and diffuse-scattered (por2). None of these patients underwent endoscopic mucosal resection, palliative resection, or preoperative chemotherapy, or had synchronous or metachronous multiple cancer in other organs. The clinicopathologic features of these patients with gastric cancer were summarized in Table 1.

**Table 1:** Correlations of miR-22 expression with the clinicopathological features of primary gastric cancer

**Features**	**No. of cases**	**miR-22 expression**		**P**
		**High**	**Low**	
**Age (years)**	98	62.8 ± 23.2	62.1 ± 23.9	NS
**Gender**				
Male	62 (63.3)	25 (40.3)	37 (59.7)	NS
Female	36 (36.7)	15 (41.7)	21 (58.3)	
**Histopathological type**				
*Intestinal type*				
pap	5 (5.1)	2 (40.0)	3 (60.0)	NS
tub1	20 (20.4)	9 (45.0)	11 (55.0)	
tub2	25 (25.5)	8 (32.0)	17 (68.0)	
*Diffuse type*				
por1	15 (15.3)	6 (40.0)	9 (60.0)	NS
por2	33 (33.7)	15 (45.5)	18 (54.5)	
**Tumor depth (pT)**				
pT1	40 (40.8)	20 (50.0)	20 (50.0)	NS
pT2	30 (30.6)	10 (33.3)	20 (66.7)	
pT3	20 (20.4)	8 (40.0)	12 (60.0)	
pT4	8 (8.2)	2 (25.0)	6 (75.0)	
**Lymph node metastasis (pN)**				
pN0	50 (51.0)	27 (54.0)	23 (46.0)	0.02
pN1	20 (20.4)	8 (40.0)	12 (60.0)	
pN2	15 (15.3)	4 (26.7)	11 (73.3)	
pN3	13 (13.3)	1 (7.7)	12 (92.3)	
**Distant metastasis (pM)**				
pM0	86 (87.8)	39 (45.3)	47 (54.7)	0.01
pM1	12 (12.2)	1 (8.3)	11 (91.7)	
**pStage**				
I	50 (51.0)	29 (58.0)	21 (42.0)	0.02
II	15 (15.3)	5 (33.3)	10 (66.7)	
III	20 (20.4)	5 (25.0)	15 (75.0)	
IV	13 (13.3)	1 (7.7)	12 (92.3)	
**Lymphatic invasion**				
Negative	45 (45.9)	17 (37.8)	28 (62.2)	NS
Positive	53 (54.1)	23 (43.4)	30 (56.6)	
**Venous invasion**				
Negative	68 (69.4)	23 (33.8)	45 (66.2)	NS
Positive	30 (30.6)	17 (56.7)	13 (43.3)	
**Hematogenous recurrence**				
Negative	78 (79.6)	32 (41.0)	46 (59.0)	NS
Positive	20 (20.4)	8 (40.0)	12 (60.0)	

Note: ‘NS’ refers to ‘no significant’.

All patients had follow-up after surgery, with X-ray examination and tumor marker assays (carcinoembryonic antigen and carbohydrate antigen 19–9) performed every 1–3 months, computed tomography performed every 3–6 months, and ultrasonography performed every 6 months. Median follow up period was 38 (range 6 to 139) months for all patients. Overall survival was defined as the period between the time of surgery and death or was censored at the last follow-up. Patients, who died of diseases not directly related to their gastric cancers or due to unexpected events, were excluded from this study.

### Quantitative RT-PCR

In order to detect the expression levels of miR-22 in gastric cancer and matched normal adjacent mucosa, quantitative RT-PCR was performed. Briefly, total RNAs, including the miRNAs, were extracted from 98 primary gastric cancer tissues and matched normal adjacent mucosa using the miRNeasy Mini Kit (Qiagen Inc., Valencia, CA, USA) according to the user’s instruction. cDNA was synthesized from 10 ng of total RNA using TaqMan™ MicroRNA hsa-miR-22 specific primer (Applied Biosystems) and a TaqMan™ MicroRNA Reverse Transcription Kit (Applied Biosystems). RNU6B was used as an internal control. The reverse transcriptase reactions contained 10 ng of total RNAs, 50 nmol/stem-loop RT primer, 1X RT buffer, 0.25 mmol/L each of dNTP, 3.3 U/μL MultiScribe reverse transcriptase, and 0.2 U/μL RNase inhibitor. The 15 μL reaction samples were incubated in GeneAmp PCR System 9700 (Applied Biosystems) for 30 min at 20°C, 30 min at 42°C, 5 min at 95°C, and then held at 4°C. Quantitative real-time PCR were performed using ABI StepOne (Applied Biosystems). The PCR conditions were initial denaturation at 95°C for 10 min, followed by 44 cycles of denaturation at 95°C for 10 sec, annealing at 56°C for 10 sec, and extension at 60°C for 10 sec. Analysis was performed by the comparative threshold cycle (Ct) method according to User Bulletin no.2 (Applied Biosystems). Each sample was examined in triplicate and the amounts of the PCR products produced were normalized to RNU6B.

### Statistical analysis

The software of SPSS version12.0 for Windows (SPSS Inc, IL, USA) and SAS 9.1 (SAS Institute, Cary, NC) was used for statistical analysis. Data were expressed as means ± standard deviation (SD). The differential expression of miR-22 between gastric cancer and matched normal adjacent mucosa was evaluated by paired sample *t* test. The *Χ*^2^ test was used to analyze the relationship between miR-22 expression and the clinicopathologic characteristics. The Kaplan–Meier method was used for survival analysis, and differences in survival were estimated using the log-rank test. Prognostic factors were examined by univariate and multivariate analyses (Cox proportional hazards regression model). Differences were considered statistically significant when *p* was less than 0.05.

## Results

### Reduced expression of microRNA-22 is associated with advanced clinicopathologic characteristics of patients with gastric cancer

**Figure 1:**
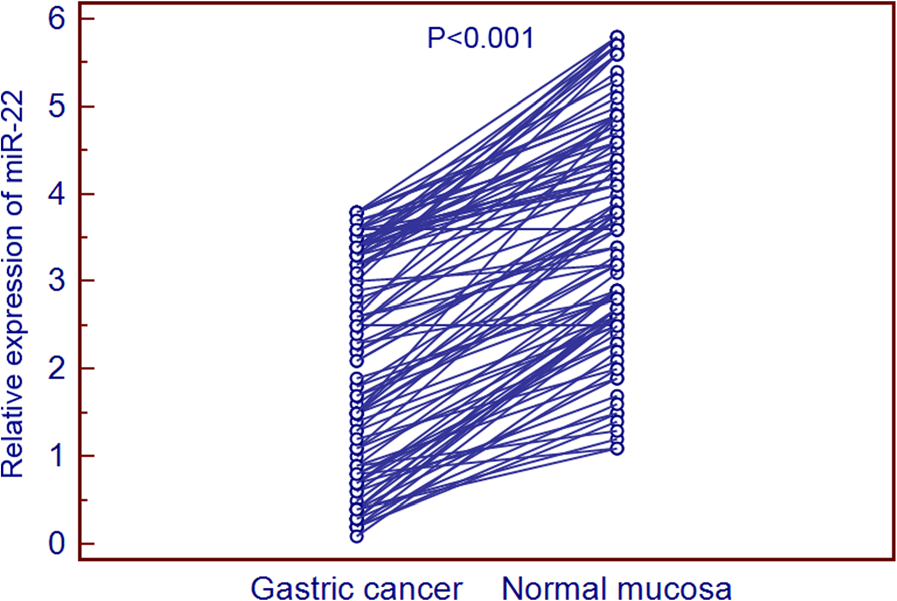
**Expression levels of miR-22 in 98 pairs of gastric cancer tissues and normal adjacent gastric mucosa.** miR-22 expression was significantly downregulated in gastric cancer tissues when compared with normal adjacent mucosa (P < 0.001).

Quantitative RT-PCR was performed to detect the differential expression of miR-22 in 98 pairs of gastric cancer and matched normal adjacent mucosa tissues normalized to RNU6B. As a result, miR-22 expression in gastric cancer was significantly lower than that in normal adjacent mucosa (mean ± SD: 2.1 ± 1.2 vs. 3.6 ± 1.3, P < 0.001, Figure 1).

The 98 patients with gastric cancer were classified into two groups according to the median expression level of miR-22 (2.2, normalized to RNU6B) as determined by quantitative RT-PCR. Of 98 patients with gastric cancer, 58 (59.2%) were placed in the low miR-22 expression group and 40 (40.8%) were placed in the high miR-22 expression group. The association between clinicopathologic features and miR-22 expression was summarized in Table 1. Tumors with low miR-22 expression had greater extent of lymph node metastasis (P = 0.02) and distant metastasis (P = 0.01), and were at a worse stage (P = 0.01) than the tumors with high miR-22 expression.

### Reduced expression of microRNA-22 confers poor prognosis in patients with gastric cancer

**Figure 2:**
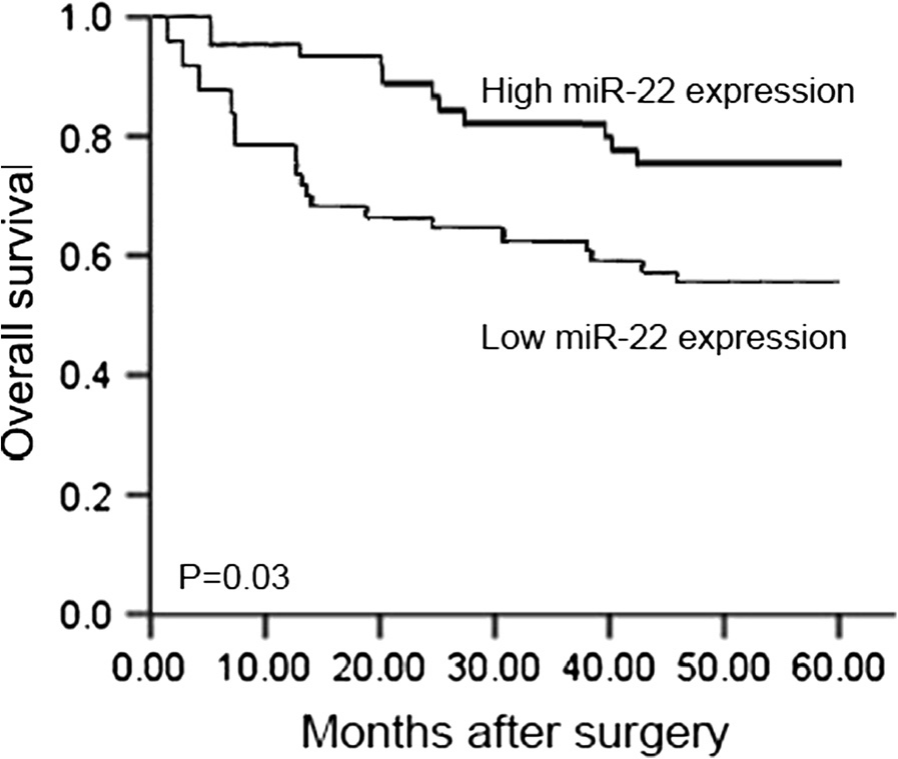
**Postoperative 5-year survival curves of patients according to the expression of miR-22.** The gastric cancer patients with low miR-22 expression had shorter overall survival than those with high miR-22 expression (P = 0.03).

All 98 patients with gastric cancer received follow-up after surgery. No patient died of postoperative complications within 30 days at the beginning of the study period. The 5-year survival rate of patients with tumors with high miR-22 expression was 82.5% (33/40), whereas the rate for patients with low miR-22 expression was 58.6% (34/58). Thus, the gastric cancer patients with low miR-22 expression had shorter overall survival than those with high miR-22 expression (P = 0.03, Figure 2).

The univariate and multivariate analyses were also performed to identify factors related to patient prognosis. As shown in Table 2, the univariate analysis showed that the depth of tumor invasion (P = 0.006), lymph node metastasis (P = 0.001), venous invasion (P = 0.03), tumor stage (P = 0.03) and miR-22 expression (P = 0.03) were significantly related to postoperative survival. Moreover, the multivariate regression analysis indicated that the depth of invasion (P = 0.01), lymph node metastasis (P = 0.01) and miR-22 expression (P = 0.04) were independent prognostic factors for patients with gastric cancer.

**Table 2:** Univariate and multivariate analyses of prognostic factors in gastric cancer

**Independent factors**	**Univariate P**	**Multivariate P**	**Hazard ratio**	**95% confidence interval**
**Tumor depth (pT)**				
pT1 and pT2/pT3 and pT4	0.006	0.01	3.8	1.0 ~ 7.2
**Lymph node metastasis (pN)**				
Negative/positive	0.001	0.01	4.1	1.2 ~ 8.9
**Venous invasion**				
Negative/positive	0.03	NS	1.8	0.5 ~ 3.1
**pStage**				
I and II and III and IV	0.03	NS	1.2	0.09-2.3
**miR-22 expression**				
Negative/positive	0.03	0.04	2.2	0.6 ~ 5.2

Note: ‘NS’ refers to ‘no significant’.

## Discussion

Accumulating evidences have demonstrated that miRNAs play important roles in various physiological and pathological processes, and have a robust association with carcinogenesis. miRNAs have been considered to be novel biomarkers for various cancers. Among human miRNAs, miR-22 is located at a fragile cancer-relevant genomic region in chromosome 17 (17p13.3), and is mapped to an exon of the C17orf91 gene [24]. This miRNA plays unique roles in specific cell types. For example, it regulates PPAR-alpha and BMP7 signaling pathways in human chondrocytes [25], and the differentiation of a monocyte cell line [26]. Recent studies have demonstrated that miR-22 is deregulated in many types of cancers and is involved in various cellular processes related to carcinogenesis, including cell growth, apoptosis, motility, and cell cycle. Zhang et al. [16] indicated that miR-22 was downregulated in hepatocellular carcinoma and had considerable potential in identification of the prognosis; Xiong et al. [17] found that miR-22 was frequently downregulated in ERα-positive human breast cancer cell lines and clinical samples; Li et al. [18] identified miR-22 as a potential metastasis-inhibitor in ovarian cancer; Yamakuchi et al. [19] found that miR-22 expression in human colon cancer was lower than in normal colon tissue, and it might have an anti-angiogenic effect in this cancer; Ling et al. [20] observed the downregulation of miR-22 in lung cancer tissues and lung cancer cell lines, and also suggested that miR-22 might exhibit excellent anti-lung cancer activity in vitro and in vivo. All these studies suggest that miR-22 may act as a tumor suppressor. In contrast, Poliseno et al. [21] showed that miR-22 was aberrantly overexpressed in human prostate cancer; Liu et al. [22] have reported that miR-22 might act as a micro-oncogene in transformed human bronchial epithelial cells induced by anti-BPDE. These controversial findings of miR-22 in cancer development suggest the diverse roles of miR-22 in different types of cancer. In the present study, we confirmed that miR-22 expression was frequently reduced in gastric cancer tissues than in their normal adjacent mucosa. Moreover, the downregulation of miR-22 was found to be more frequently occurred in gastric cancer tissues with great extent of lymph node and distant metastases, and with an advanced stage.

To our knowledge, the invasion and metastasis of tumor cells are major causes of mortality in cancer patients. Therefore, the potential value of miR-22 as a prognostic marker is of interest. Yet, there has been only one study that has attempted to identify the prognostic value of miR-22 for hepatocellular carcinoma. Using 160 primary hepatocellular carcinoma cases, Zhang et al. [16] found that low miR-22 expression correlated with poor overall survival. In line with this finding, we analyzed not only the Kaplan-Meier survival curve but also applied Cox multivariate analysis to clarify the prognostic value of miR-22 in gastric cancer. Notably, we demonstrated that low miR-22 expression in gastric cancer tissues significantly correlated with poorer overall survival. Furthermore, in Cox multivariate analysis, miR-22 expression in gastric cancer tissues showed a significant association with overall survival.

In conclusion, our data offer the convincing evidence that the reduced expression of miR-22 was significantly associated with malignant development of gastric cancer and may be a novel prognostic marker of this disease. miR-22 might have potentials in the application of cancer therapy for patients with gastric cancer. However, our study is limited by the number of study cases with relatively small subgroups. Further investigations with a larger number of cases would allow us to evaluate miR-22 in a variety of clinical settings and help us better understand its unique role in cancer progression.

## Competing interests

The authors declare that they have no competing interests.

## Authors’ contributions

WW and FL designed the study, carried out the experiments and drafted the manuscript; YZ, YT, QY and XG participated in the experiments and data analysis. All authors read and approved the final manuscript.
